# ProMix-DGNet: A Process-Aware Spatiotemporal Network for Sintering System Prediction

**DOI:** 10.3390/s26061953

**Published:** 2026-03-20

**Authors:** Zhili Zhang, Yuxin Wan, Liya Wang, Jie Li

**Affiliations:** 1 College of Science, North China University of Science and Technology, Tangshan 063210, China; zhangzhili@stu.ncst.edu.cn; 2 College of Metallurgy and Energy, North China University of Science and Technology, Tangshan 063210, China; wanyuxin@stu.ncst.edu.cn; 3 Hebei Key Laboratory of Data Science and Application, North China University of Science and Technology, Tangshan 063210, China; 4 The Key Laboratory of Engineering Computing in Tangshan City, North China University of Science and Technology, Tangshan 063210, China; 5 Hebei Engineering Research Center for the Intelligentization of Iron Ore Optimization and Ironmaking Raw Materials Preparation Processes, North China University of Science and Technology, Tangshan 063210, China; 6 Yanzhao Iron and Steel Laboratory, Tangshan 063210, China

**Keywords:** Industrial Internet of Things, sintering process, spatiotemporal graph neural networks, dynamic graph constructor, large time-delay system

## Abstract

Multistep-ahead prediction of critical states in the iron ore sintering process is essential for maintaining production stability, enhancing energy efficiency, and reducing industrial emissions. However, large time delays, strong coupling, and condition drifts challenge existing spatiotemporal graph neural networks (STGNNs). This paper proposes Process-aware Mixed Dynamic Graph Network (ProMix-DGNet), which integrates a Decoupled Two-Stream Topology Learning mechanism—fusing Adaptive Static Graph with a Radial Basis Function (RBF)-driven Dynamic Graph Constructor—to ensure robust spatial modeling under high-noise conditions. Furthermore, Process-View Global Mixer explicitly captures long-range process coupling across the entire sintering strand, overcoming the receptive field limitations of traditional graph convolutions. In the decoding phase, a future control-informed module utilizes a bidirectional Long Short-Term Memory (BiLSTM) and a global mixer to align known future control setpoints with the system’s spatial topology. These features are integrated via a gated residual mechanism that dynamically modulates the interaction between control intents and historical representations. Extensive experiments conducted on two real-world industrial datasets, Sinter-A and Sinter-B, demonstrate that ProMix-DGNet consistently outperforms mainstream baselines across multiple metrics, including Mean Absolute Error (MAE) and Root Mean Square Error (RMSE). The results verify the model’s higher accuracy and robustness in complex large-time-delay systems, offering a reliable framework for the intelligent monitoring and closed-loop optimization of sintering process.

## 1. Introduction

Iron ore sintering is the primary raw material supply process for blast furnace ironmaking. The yield and quality of the finished sinter directly determine the production efficiency and energy consumption of the subsequent ironmaking stages [1]. Simultaneously, sintering is one of the most significant sources of energy consumption and pollutant emissions in the steel industry [2]. Therefore, achieving precise, multistep-ahead prediction of the sensor network—which serves as the data foundation for deriving key state variables such as wind box temperature distribution, exhaust gas composition, and the burn-through point (BTP)—is essential for process stability. By accurately forecasting the spatiotemporal evolution of these process variables, the model provides the necessary high-fidelity inputs for building closed-loop intelligent control systems and reducing industrial emissions. However, the sintering environment is characterized by harsh on-site conditions, highly coupled process parameters, and significant non-linearity and time-varying dynamics [3]. These factors make traditional modeling methods based on thermodynamics or chemical reaction mechanisms difficult to adapt to fluctuating production conditions. To address these issues, deep learning, which integrate massive operational data and possess strong nonlinear fitting capabilities, have emerged as a research hotspot for achieving intelligent perception and coordinated control in sintering [4].

Early predictions of the sintering process primarily relied on time series analysis methods (such as ARIMA) or traditional Recurrent Neural Networks (RNN, LSTM). Although these methods can capture the temporal dependencies of individual sensors [5], they ignore the topological relationships between different spatial locations on the sintering machine, such as the wind box and flues distributed along the length of the sintering strand. In recent years, Spatiotemporal Graph Neural Networks (STGNN) has achieved significant success in domains like traffic flow prediction [6], environmental monitoring [7], energy management [8], and brain network analysis [9], and are now being introduced into industrial process modeling. By modeling sensors as graph nodes and employing Graph Convolutional Network (GCN) to capture spatial dependencies combined with Temporal Convolutional Network (TCN) or Recurrent Neural Network (RNN) for temporal features, these models effectively extract spatiotemporal correlations. For instance, Graph WaveNet [10] and MTGNN [11] have addressed the issue of missing predefined graph structures through adaptive adjacency matrices, which can be further enhanced by incorporating semantic knowledge [12] or physics-informed constraints [13] to improve the modeling of complex dynamic systems.

Despite their potential, applying existing STGNN methods to complex sintering processes faces three critical challenges that limit prediction accuracy.

First, the robustness of dynamic graph learning against industrial stochastic noise is insufficient. The sintering process operates under harsh environments that generate highly non-stationary and high-amplitude noise, necessitating a graph structure capable of stable evolution. However, recent authoritative studies highlight that dynamic spatiotemporal models are intrinsically vulnerable to structural perturbations and such noise [14,15]. This stochastic noise frequently triggers a “dual noise effect” in industrial datasets, leading to severe topology degradation, sample indistinguishability, and feature collapse [16]. Although existing methods utilize attention mechanisms [17,18] or recursive structures [19,20] to capture dynamics, they often struggle here: attention models are highly susceptible to outliers, while recursive approaches suffer from temporal inertia that excessively smooths historical states and suppresses the detection of abrupt transient changes [21]. Furthermore, methods relying on discrete Top-K selection introduce non-differentiability and potential numerical oscillations during training [22]. Achieving a stable yet sensitive dynamic graph under high-variance industrial conditions remains a challenge in spatiotemporal modeling.

Second, the constrained local receptive field of traditional GCN severely limits their ability to capture macroscopic spatial coupling and large time delays. Iron ore sintering is a continuous, long-distance pipeline process where thermal state changes in the ignition section at the head significantly affect the discharge quality at the tail with massive transport delays. However, traditional GCN primarily extract local features by aggregating information from immediate neighbors, which inherently fails to capture such complex macroscopic spatiotemporal coupling [23]. While stacking multiple GCN layers can theoretically expand this local vision, it frequently triggers the over-smoothing problem, where node representations become indistinguishable and lose their task-specific utility [10,24]. Furthermore, attempting to capture global dependencies by generating dense adaptive adjacency matrices fundamentally incurs computational complexity, severely restricting its scalability over extended physical networks [25].

Finally, future control variables are underutilized. In industrial control systems, future setpoints—such as strand speed and main exhaust fan frequency—are often known a priori as deterministic feedforward signals. Existing spatiotemporal models typically treat these variables by simple concatenation with historical features. This approach fails to account for the dynamic driving effect exerted by temporal evolution of control signals, ignoring how these trends actively steer the future state of the system over time [26].

To address these challenges, this paper proposes the Process-aware Mixed Dynamic Graph Network (ProMix-DGNet). Architecturally, it integrates a decoupled two-stream topology learning mechanism with a Process-View Global Mixer to harmonize localized diffusion with macroscopic process constraints. Specifically, the Global Mixer works in tandem with graph convolutions to refine localized spatial representations, while a bidirectional Long Short-Term Memory (BiLSTM)-based decoding module further aligns these patterns with predetermined future operational signals. This integrated design enables the model to maintain predictive stability under high-variance industrial conditions while remaining responsive to active control interventions.

The main contributions of this paper are summarized as follows:

- Unlike mainstream adaptive STGNN, we propose a dynamic graph constructor driven by a Radial Basis Function (RBF) kernel. By using absolute Euclidean distance, this design provides an exponential decay effect that truncates extreme industrial outliers, providing a robust topological inductive bias against non-stationary condition drifts. To ground this data-driven process, we introduce a static graph based on physical process lags, preventing the topological collapse often seen in unconstrained adaptive networks.
- To handle the large time delays of the sintering strand, we introduce the Process-View Global Mixer as a vital complement to traditional GCN. This module establishes a high-speed information channel that processes the localized features extracted by graph convolutions, effectively bypassing their inherent receptive field limitations. By anchoring global interactions to the machine’s physical layout with linear computational complexity (O(N)), it captures long-range thermodynamic couplings across the entire strand without the over-smoothing issues of deep GCN stacks.
- We bridge the gap between predictive modeling and industrial feedforward control by designing a Future Control-Informed Decoding Mechanism. Instead of simple feature concatenation, it formulates the prediction as a condition-modulated process. This allows the network to proactively translate known future control matrices into system-level responses, ensuring rapid adaptation to regime switches under harsh operational transitions.
- Extensive experimental validation on real-world datasets demonstrates that ProMix-DGNet consistently achieves superior performance across the vast majority of error metrics compared to recent state-of-the-art baselines. Notably, on the highly volatile Sinter-B dataset, the model reduces the Root Mean Square Error (RMSE) by 2.39% (from 976.166 to 952.832) compared to the currently strongest baseline, Towards Expressive Spectral–Temporal Graph Neural Networks (TGGC). Paired *t*-tests confirming high statistical significance (*p* < 0.001) verify that these improvements are driven by our architectural design.

## 2. Related Work

### 2.1. Evolution of Sintering Process Modeling

Research on sintering process modeling has undergone a significant paradigm shift from mechanism-based analysis to data-driven intelligence. Early approaches primarily relied on physicochemical equations or Computational Fluid Dynamics (CFD) simulations to describe thermal states [27]. However, the prohibitive computational costs and complex boundary conditions of these methods often fail to meet the real-time monitoring requirements of modern industry. As industrial data collection has expanded, the focus shifted toward data-driven paradigms, initially utilizing statistical methods (e.g., PCA) or traditional machine learning (e.g., SVM, XGBoost) for soft sensing [28]. Subsequently, deep learning models like LSTMs and CNNs were introduced to capture long-range temporal dependencies and grid-based local spatial features, respectively. Unlike Long-term Time Series Forecasting (LTSF), which focuses on single-dimensional temporal patterns, Spatiotemporal Forecasting (STF) offers a higher-dimensional paradigm that simultaneously deconstructs the crisscross spatial dependencies and physical transmission between sensor nodes while modeling nonlinear temporal dynamics.

### 2.2. Spatiotemporal Modeling in Industrial IoT and Limitations of STGNNs

In modern industrial IoT ecosystems, the data pipeline is divided into perception and application layers. At the perception layer, advancements in Wireless Sensor Network (WSN) integrate deep learning for reliable data collection. Protocols such as DL-HEED [29] and QPSODRL [30] employ graph neural networks and reinforcement learning to optimize node clustering and dynamic routing. Continuous monitoring within these systems generates large volumes of coupled spatiotemporal data. The challenge at the application layer is mining this network data for process optimization.

STGNNs serve as a primary architecture for this task. However, existing STGNNs exhibit limitations when applied to industrial processes such as sintering. Early models, such as STGCN [31] and DCRNN [19], typically relied on predefined graph structures based on physical distance or prior knowledge. However, these models were originally designed for traffic networks with fixed physical topology. In contrast, the sintering process involves invisible, time-varying thermochemical couplings that cannot be predefined by a static Euclidean graph, making these methods susceptible to concept drift.

While recent advancements like MTGNN [11] and AGCRN [32] introduced graph self-learning mechanisms to automatically mine latent variable associations from data, these standard adaptive STGNN primarily rely on dot-product or cosine similarity between unconstrained node embeddings. In highly volatile industrial environments, such magnitude-invariant metrics are highly sensitive to high-amplitude stochastic noise and extreme outliers, frequently leading to dynamic topology collapse. Furthermore, purely data-driven adaptive graphs lack physical constraints, making them prone to overfitting transient perturbations rather than preserving the sequential causality of the continuous manufacturing process.

### 2.3. Global Dependency Modeling and Feedforward Control Fusion

Despite these improvements, most state-of-the-art STGNNs remain fundamentally based on GCN operators. Consequently, they are constrained by a local receptive field, leading to insufficient perception capabilities when modeling the global dependencies of device nodes spanning large-scale time-delays. In a typical sintering strand, the raw mix on the pallets travels from the ignition hood to the discharge end over 45–60 min. This temporal delay is coupled with complex wind box pressure fluctuations and the downward migration of the combustion zone. The shallow propagation of local GCN operators struggles to capture the long-range spatial correlations inherent in such sequential manufacturing processes.

To overcome the limitations of local GCN, researchers have drawn inspiration from recent advances in the LTSF domain. Studies such as LTSF-Linear [33] and iTransformer [34] have questioned the robustness of self-attention mechanisms in high-noise industrial environments, noting that their permutation-invariant nature may lead to the loss of critical temporal information under stochastic disturbances. In contrast, MLP-based architectures, such as TS-Mixer [35], have demonstrated that global features can be efficiently extracted through fully connected Token-mixing or Channel-mixing layers without relying on attention, offering structural robustness for fixed sensor topologies. Furthermore, existing spatiotemporal models often overlook control causality, focusing primarily on historical data backtracking. Recent research emphasizes that integrating feedforward features—future control setpoints—can reflect system dynamics more proactively [26]. For instance, the temperature distribution across wind box is not just a result of local reactions, but a cumulative effect of the material layer’s permeability and the suction pressure established earlier in the process. Therefore, deeply integrating these global feedforward characteristics into a spatiotemporal framework to overcome large time delays remains a critical unresolved issue in current research.

## 3. Problem Definition

The underlying topology of the sintering system is modeled as a directed graph G=(V,E,A), where V represents the set of variable nodes with |V|=N, E is the set of edges, and $A\in {\mathbb{R}}^{N\times N}$ is the adjacency matrix. Let $V_{ctrl}\subset V$ denote the predefined set of exogenous control nodes, and $V_{target}\subset V$ denote the set of endogenous target nodes, such that $V_{ctrl}\cap V_{target}=\emptyset$ with sizes $|V_{ctrl}|=N_{ctrl}$ and $|V_{target}|=N_{target}$.

Let $X_{t-P+1:t}\in {\mathbb{R}}^{P\times N\times D_{in}}$ denote the historical observation matrix; P is the look-back window, and $D_{in}$ is the input feature dimension comprising the historical states of all N nodes. Let $C_{t+1:t+Q}\in {\mathbb{R}}^{Q\times N_{sp}\times D_{fut}}$ denote the future control matrix over the prediction horizon Q. This matrix serves as the known exogenous input, embedding the predetermined trajectories of the control nodes $v_{i}\in V_{ctrl}$, while the unobserved features of other nodes are mathematically masked.

Given the historical observations $X_{t-P+1:t}$, the future exogenous conditions $C_{t+1:t+Q}$, and the graph topology G, the objective is to learn a spatiotemporal mapping function $F_{\Theta }$ to forecast the future trajectories exclusively for the target nodes ${\hat{Y}}_{t+1:t+Q}\in {\mathbb{R}}^{Q\times N_{target}\times 1}$:

(1) $${\hat{Y}}_{t+1:t+Q}=F_{\theta }(X_{t-P+1:t},C_{t+1:t+Q};G)$$

## 4. Method

### 4.1. Overall Architecture

**Figure 1:**
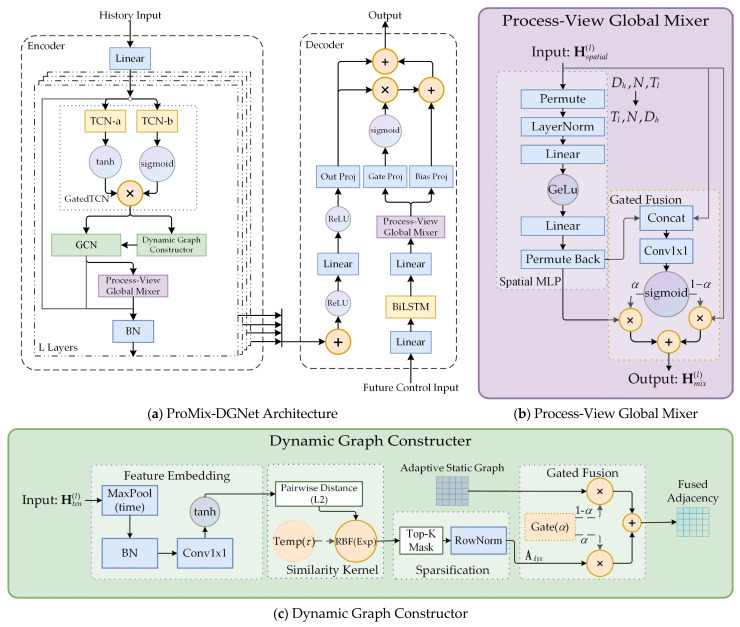
The overall architecture of ProMix-DGNet.

The proposed model, Process-aware Mixed Dynamic Graph Network (ProMix-DGNet), utilizes an encoder–decoder architecture designed to integrate multiscale temporal patterns with decoupled spatial dependencies. The architecture is organized into three main components: an Input Projection Layer, L Stacked Process-Aware Spatiotemporal Encoders, and a Future Control-Informed Decoder. The integrated structure and its internal modules are illustrated in Figure 1. Specifically, Figure 1a outlines the overall pipeline, while Figure 1b and Figure 1c provide detailed views of the Process-View Global Mixer and the Dynamic Graph Constructor, respectively.

To address the dimensional ($D_{in}$) discrepancies in historical observations across different factory datasets, the model first employs an Input Projection Layer to map the raw input into a unified high-dimensional hidden space. Given the historical observation matrix $X_{t-P+1:t}\in {\mathbb{R}}^{P\times N\times D_{in}}$, we apply a 1×1 convolutional layer over the feature channel dimension:

(2) $$H^{\left(0\right)}=\mathrm{Permute}(X_{t-P+1:t})*W_{in}+b_{in}$$

where the Permute operation realigns the data to a channel-first format to facilitate the subsequent stacked encoding operations. The symbol ∗ denotes the channel-wise convolution operation. $W_{in}\in {\mathbb{R}}^{D_{in}\times D_{h}}$ and $b_{in}\in {\mathbb{R}}^{D_{h}}$ represent the learnable weight and bias parameters, respectively. This linear mapping ensures that heterogeneous physical signals are standardized into a cohesive representation $H^{\left(0\right)}\in {\mathbb{R}}^{D_{h}\times N\times P}$ for the subsequent encoding stages.

The core of ProMix-DGNet consists of *L* stacked blocks, each partitioned into two functional stages that mirror the physical spatiotemporal propagation of the sintering strand. The first stage establishes the structural foundation through temporal trend and topology learning. Within each block, a gated TCN condenses the historical observation matrix into a macro-dynamic snapshot. Utilizing these layer-specific temporal features, a two-stream graph topology learning module generates a hybrid graph fusing static physical anchors with data-driven dynamic metrics. The second stage facilitates information exchange through spatial interaction and global mixing. Building upon the learned topology, it performs localized spatial diffusion via GCN to capture fine-grained sensor couplings. Subsequently, the Process-View Global Mixer overcomes physical distance limitations by aggregating localized features into a system-level representation, which reflects the global operational state of the entire sintering machine.

In the output stage, the model uses a multi-scale skip connection architecture to converge representations from different depths and introduces a Future Control-Informed Decoding Mechanism. This employs BiLSTM to extract temporal dependencies from future control setpoints. The process-view global mixer aligns these signals with the propagation laws of the physical topology. These future contexts are dynamically integrated with historical representations via a gated residual mechanism, ensuring rapid adaptation to regime switches under non-stationary conditions.

### 4.2. Stage I: Temporal Trend Encoding and Topology Learning

#### 4.2.1. Gated Temporal Convolutional Network

To capture the temporal evolution of the sintering process, we use gated TCN as the frontend encoder. The gating mechanism handles the non-stationary and noisy nature of industrial sensor data by prioritizing salient thermodynamic trends over high-frequency fluctuations.

Within each encoder layer *l* (l∈{1,…,L}), the input hidden state is denoted as $H_{mix}^{\left(l-1\right)}$ (with the initial projection specifically defined as $H_{mix}^{\left(0\right)}\equiv H^{\left(0\right)}$). This state is transformed into a localized temporal representation $H_{tcn}^{\left(l\right)}$ via a dual-branch dilated causal convolution:

(3) $$H_{tcn}^{\left(l\right)}=\tanh(\Theta_{f}*H_{mix}^{\left(l-1\right)}+b_{f})⊙\sigma (\Theta_{g}*H_{mix}^{\left(l-1\right)}+b_{g})$$

Here, ∗ denotes the convolution operation, while $\Theta_{f},\Theta_{g},b_{f},b_{g}$ are learnable parameters. The distinct activation functions for the two branches, denoted as TCN-a and TCN-b, are designed to handle the non-stationary and noisy nature of industrial sintering data. The TCN-a branch ($\Theta_{f}$) acts as the primary feature extractor utilizing the tanh activation function, bounding the output within [−1,1]. The TCN-b branch ($\Theta_{g}$) functions as a dynamic information filter using the sigmoid activation function (σ), constraining its output to the range [0,1]. Through element-wise multiplication, this gating mechanism determines the proportion of the extracted features allowed to pass to the next layer.

To map the physical observation window P=12 into a representative macro-dynamic snapshot, we implement a temporal squeezing strategy. Configuring the dilation factors as $d_{l}\in \{1,2,4,8\}$ with a kernel size k=2 and applying initial zero-padding aligns the input with the network’s receptive field. Cascading four encoder blocks progressively shrinks the sequence length via $T_{l}=T_{l-1}-(k-1)d_{l}$, collapsing the temporal dimension to $T_{L}=1$. The temporally filtered tensor $H_{tcn}^{\left(L\right)}\in {\mathbb{R}}^{D_{h}\times N\times 1}$ then feeds into the subsequent graph-based modules for dynamic graph constructor, spatial diffusion and global interaction.

#### 4.2.2. Decoupled Two-Stream Topology Learning

Spatial dependencies in sintering systems involve both physically fixed equipment topologies that establish long-term stable correlations and transient thermodynamic states driven by continuous operational fluctuations. To decouple and capture these distinct patterns, we propose a two-stream topology learning architecture featuring a dynamic graph constructor (Figure 1c). The first stream constructs an adaptive static graph ($A_{static}$) initialized by the predefined physical prior to model stable mechanical connections. The second stream dynamically generates a soft dynamic graph ($A_{dynamic}$) driven by real-time temporal convolutional features ($H_{tcn}^{\left(L\right)}$) to capture transient correlations. These two streams are adaptively fused to form the comprehensive spatial topology.

To embed deterministic physical mechanisms into the network, we initialize the node embedding dictionaries $E_{1},E_{2}\in {\mathbb{R}}^{N\times D_{d}}$ of $A_{static}$ using truncated singular value decomposition (SVD) on the pre-calculated physical prior matrix $A_{prior}$ (detailed in Section 5.1.3):

(4) $$A_{prior}\approx U_{d}\Sigma_{d}V_{d}^{T}$$

We initialize the embeddings as $E_{1}^{\left(init\right)}=U_{d}\Sigma_{d}^{1/2}$ and $E_{2}^{\left(init\right)}=V_{d}\Sigma_{d}^{1/2}$. We formulate the adaptive graph as:

(5) $$A_{static}=\mathrm{Softmax}(\mathrm{ReLU}(E_{1}E_{2}^{T}))$$

The ReLU activation acts as a hard filter to eliminate physically meaningless negative weights, and the SoftMax function normalizes the spatial connections. During training, backpropagation updates $E_{1}$ and $E_{2}$. This ensures the topology is initially grounded in industrial mechanisms while remaining adaptive.

While the static stream captures long-term stability, the second stream infers a dynamic graph $A_{dynamic}$ to model transient dependencies. We derive this graph from the intermediate temporal features $H_{tcn}^{\left(L\right)}\in {\mathbb{R}}^{D_{h}\times N\times T_{l}}$ produced by the gated TCN in Section 4.2.1. To extract salient spatial representations while handling the temporal dimension $T_{l}$, we apply a temporal max-pooling operation followed by a 1×1 convolution to project the features into a lower-dimensional embedding space $E_{d}\in {\mathbb{R}}^{N\times D_{d}}$:

(6) $$E_{d}=\tanh(\mathrm{MaxPool}(H_{tcn}^{\left(l\right)})W_{d}+b_{d})$$

where MaxPool(·) aggregates the most significant activations across the temporal dimension $T_{l}$, and $W_{d}\in {\mathbb{R}}^{D_{h}\times D_{d}}$ is the learnable projection weight. The tanh activation function ensures that the node embeddings are centered and bounded within [−1,1], providing a stable basis for similarity measurement. We then compute pairwise spatial similarities using an RBF kernel with a learnable temperature τ:

(7) $$S_{i,j}=\exp\left(-\frac{\|E_{i}-E_{j}{\|}_{2}^{2}}{\tau }\right)$$

This formulation introduces a distance-decay inductive bias that aligns with the physical reality of industrial sintering, where sensors operating under similar thermodynamic conditions aggregate into functional clusters. By mapping latent proximity to structural connectivity, this kernel generates a topology that reflects the thermodynamic homophily of transient spatial correlations. The learnable temperature parameter τ acts as an adaptive regulator for the dynamic receptive field. During continuous operational fluctuations, τ dynamically scales the sensitivity of the Euclidean distance metric. It tightens to capture fine-grained local similarities during stable phases and relaxes to maintain fundamental topological connectivity during high-variance condition drifts. This data-driven scaling mechanism ensures stable convergence under varying industrial noise levels.

To mitigate background noise and reduce interaction complexity from $O(N^{2})$ to O(N), we apply a top-K sparsification strategy. An indicator mask $M\in {\left\{0,1\right\}}^{N\times N}$ retains the K strongest connections for each node i, thresholded by its K-th largest similarity value $\theta_{i}$:

(8) $$M_{i,j}=\left\{\begin{array}{ll} 1, & \mathrm{if}\;S_{i,j}\in \mathrm{top}\text{-}k(S_{i,:})\\ 0, & \mathrm{otherwise} \end{array}\right.$$

We then row-normalize the masked similarity matrix to yield the dynamic adjacency matrix $A_{dynamic}\in {\mathbb{R}}^{N\times N}$:

(9) $$A_{dynamic_{i,j}}=\frac{S_{i,j}⊙M_{i,j}}{\sum_{k=1}^{N}\left(S_{i,k}⊙M_{i,k}\right)+\epsilon }$$

where ⊙ denotes element-wise multiplication and ϵ=1e−8. We integrate the global physical prior and local real-time dynamics using an adaptive gated fusion layer:

(10) $$A_{fused}=\alpha A_{static}+(1-\alpha )A_{dynamic}$$

where the learnable parameter α∈[0,1] balances the mechanism-driven prior with data-driven perturbations. We initialize α=0.5 to provide an unbiased prior, allowing the network to optimize the balance between physical consistency and transient adaptability through end-to-end backpropagation. The resulting fused graph $A_{fused}$ provides the structural basis for the subsequent spatial diffusion convolution.

### 4.3. Stage II: Spatial Interaction and Process-View Global Mixing

#### 4.3.1. Graph Diffusion Convolution

Following the temporal snapshots and fused topology derived in Stage I, this module aggregates spatial features to capture localized node interactions. Modeling spatial dependencies as a stochastic diffusion process translates the sequential material flow and thermodynamic evolution into a directed message-passing mechanism on the graph.

To account for asymmetric spatial dependencies in the sensor network, we define three transition matrices $P=\{P_{fwd},P_{bwd},P_{dyn}\}$ to support the convolution. The forward transition matrix $P_{fwd}$, defined as $A_{s}/\mathrm{rowsum}(A_{s})$, represents downstream transmission of features along the primary operational direction. The backward transition matrix $P_{bwd}$, defined as $A_{s}^{⊤}/\mathrm{rowsum}(A_{s}^{⊤})$, captures reverse structural feedback loops within the network. The dynamic matrix $P_{dyn}$, derived from the fused graph $A_{fused}$, provides data-driven support to capture transient correlations under fluctuating conditions.

Given the temporal representation $H_{tcn}^{\left(l\right)}$ at the l-th layer, the spatial diffusion convolution is computed as:

(11) $$H_{spatial}^{\left(l\right)}=\sum_{P\in P}\sum_{k=0}^{S}P^{s}H_{tcn}^{\left(l\right)}W_{P,s}+b_{gc}$$

where *S* denotes the diffusion step, and $P^{s}$ represents the s-th power of the transition matrix, simulating the multi-hop propagation of features. The variables $H_{tcn}^{\left(l\right)},H_{spatial}^{\left(l\right)}\in {\mathbb{R}}^{D_{h}\times N\times T_{l}}$ denote the input and output spatiotemporal representations. The parameters $W_{P,s}\in {\mathbb{R}}^{D_{h}\times D_{h}}$ and $b_{gc}\in {\mathbb{R}}^{D_{h}}$ are learnable filter weights and bias vectors.

This multi-hop message-passing mechanism allows each sensor node to aggregate information from its physically and operationally connected neighborhood. Integrating directional structural priors with dynamic metrics extracts localized spatiotemporal features, providing a base for the subsequent system-level global interaction.

#### 4.3.2. Process-View Global Mixer

While GCN captures localized features, the sintering process is equally governed by macroscopic thermodynamic dependencies across the entire strand. To capture these long-range constraints and the absolute positional semantics of the fixed pipeline, we propose Process-View Global Mixer (Figure 1b). By tokenizing the spatial dimension, this module treats each sensor’s full time series as a distinct process node. This node-centric mixing allows the model to learn the functional roles of specific process points via direct linear mappings, effectively anchoring global interactions to the machine’s physical layout.

Given the localized spatiotemporal features $H_{spatial}^{\left(l\right)}\in {\mathbb{R}}^{D_{h}\times N\times T_{l}}$, we apply layer normalization and perform dimension transposition to prepare for global interaction:

(12) $$E_{token}^{\left(l\right)}=\mathrm{Permute}\left(\mathrm{LayerNorm}(H_{spatial}^{\left(l\right)})\right)$$

where $E_{token}^{\left(l\right)}\in {\mathbb{R}}^{T_{l}\times D_{h}\times N}$ represents the collection of *N* process-node tokens. In this node-centric latent space, each token encapsulates the complete temporal evolution and hidden feature channels of an individual physical sensor.

The core of the global interaction is a dual-layer feed-forward network operating directly on the spatial node dimension *N*:

(13) $$H_{mixed}^{\left(l\right)}=\mathrm{GeLU}(E_{token}^{\left(l\right)}W_{1}+b_{1})W_{2}+b_{2}$$

where $W_{1}\in {\mathbb{R}}^{N\times rN}$ and $W_{2}\in {\mathbb{R}}^{rN\times N}$ are learnable weight matrices, and r is a configurable expansion ratio. This expansion ratio projects the features into a higher-dimensional latent space, enhancing the model’s non-linear capacity to capture complex global dependencies. This spatial multilayer perceptron implicitly establishes a fully connected topological view. It grants each sensor a global receptive field and models the inherent stability of the sintering thermodynamic field. The final permutation restores the tensor to its original shape ${\mathbb{R}}^{D_{h}\times N\times T_{l}}$.

To ensure stable integration, an adaptive gated mechanism fuses the mixed features $H_{mixed}^{\left(l\right)}$ with the original localized features:

(14) $$G^{\left(l\right)}=\sigma ({\mathrm{Conv}}_{1\times 1}([H_{spatial}^{\left(l\right)};H_{mixed}^{\left(l\right)}]))$$

(15) $$H_{mix}^{\left(l\right)}=G_{mix}⊙H_{mixed}^{\left(l\right)}+(1-G_{mix})⊙H_{spatial}^{\left(l\right)}$$

where σ(·) denotes the sigmoid activation function, and [·;·] represents channel concatenation. This gated structure balances the mechanism-driven local diffusion of heat and material flow with the data-driven global perturbations governed by macroscopic thermodynamic constraints. Using the physical prior of a fixed node quantity across the sensor network, Process-View Global Mixer formulates global interactions with a linear computational complexity of O(N). This design enhances inference efficiency, providing a scalable solution for capturing long-range dependencies in industrial systems characterized by transport delays.

### 4.4. Future Control-Informed Decoding Mechanism

Industrial sintering is an intervention-driven process where the future thermodynamic state depends heavily on predetermined operational control signals, such as adjustments to strand speed or ignition temperature. To translate these future operational intents into system-level thermodynamic responses, we introduce a future control-informed decoding mechanism that formulates multi-step prediction as a condition-modulated generative process.

To preserve multi-scale spatiotemporal representations and facilitate stable gradient propagation across the deep architecture, we aggregate the intermediate latent states from all *L* spatiotemporal blocks. These skip-connections are extracted immediately after gated TCN, denoted as $H_{tcn}^{\left(l\right)}$. This specific extraction point preserves the unadulterated multi-resolution temporal momentum captured by the dilated convolutions, ranging from short-term localized fluctuations to long-term macroscopic trends. It also prevents spatial over-smoothing and retains fine-grained, node-specific features. A pointwise convolution standardizes the channel dimensions of these temporal features before aggregating them via a skip-connection framework:

(16) $$H_{agg}=\sum_{l=1}^{L}{\mathrm{Conv}}_{1\times 1}^{skip}(H_{tcn}^{\left(l\right)})$$

Causal dilated convolutions effectively summarize the historical receptive field into a compact temporal state. To project this aggregated representation $H_{agg}\in {\mathbb{R}}^{D_{h}\times N\times 1}$ into the target forecasting horizon *Q*, a non-linear prediction head composed of a two-layer pointwise (1×1) convolution network with intermediate ReLU activations is employed. The first pointwise convolution transforms the hidden features, and the second convolution maps them to the future time steps. This process is defined as:

(17) $$H_{out}=\mathrm{ReLU}(W_{out1}*\mathrm{ReLU}(H_{agg})+b_{out1})$$

(18) $${\hat{Y}}_{base}=W_{out2}*H_{out}+b_{out2}$$

where ∗ denotes the pointwise convolution operation, $W_{out1}\in {\mathbb{R}}^{D_{end}\times D_{h}}$ and $W_{out2}\in {\mathbb{R}}^{Q\times D_{end}}$ represent the learnable weight matrices, and $b_{out1},\;b_{out2}$ are the corresponding bias vectors. The resulting tensor ${\hat{Y}}_{base}\in {\mathbb{R}}^{Q\times N\times 1}$ serves as the unconditioned historical baseline prediction, representing the expected system evolution assuming zero future external interventions.

Concurrently, the network is conditioned on the exogenous future control matrix $C_{t+1:t+Q}$. As defined in Section 3 and pre-processed via zero-masking during dataset construction (Section 5.1.3), this matrix encapsulates the predetermined future trajectories strictly for the control nodes $V_{ctrl}$ while maintaining global topological consistency. To extract the operational intent, BiLSTM processes the condition tensor independently across the *N* physical nodes:

(19) $$E_{temp}=\mathrm{Linear}(\mathrm{BiLSTM}(C_{t+1:t+Q}))$$

where $E_{temp}\in {\mathbb{R}}^{D_{emb}\times N\times Q}$ represents the temporally encoded control features. Industrial control commands possess strict temporal continuity and local momentum. The recurrent formulation of the BiLSTM preserves this sequential order and avoids the quadratic computational overhead over short-horizon localized trajectories (*Q* steps), serving as a lightweight localized temporal extractor. Given the strong thermodynamic coupling in sintering, localized actuator perturbations inevitably induce global state transitions. To model this spatial propagation, we apply Process-View Global Mixer to $E_{temp}$, translating localized temporal commands into global state perturbations and yielding the spatiotemporal-aware control embedding $E_{ctrl}\in {\mathbb{R}}^{D_{emb}\times N\times Q}$.

To impose future operational controls onto the predictions derived from historical data, we formulate the integration as a conditional feature modulation process. An adaptive gated residual mechanism dynamically modulates the historical baseline based on the spatiotemporal control embeddings. The control embedding $E_{ctrl}$ generates a temporal–spatial gate mask $G_{mask}$ and a bias correction term $B_{corr}$:

(20) $$G_{mask}=\sigma (E_{ctrl}W_{mask}+b_{mask})$$

(21) $$B_{corr}=E_{ctrl}W_{corr}+b_{corr}$$

where $G_{mask},B_{corr}\in {\mathbb{R}}^{Q\times N\times 1}$ represent the dynamic gate mask and bias correction tensor, respectively. The final predictive output ${\hat{Y}}_{t+1:t+Q}$ modulates the historical baseline with these control-derived parameters:

(22) $${\hat{Y}}_{t+1:t+Q}={\hat{Y}}_{base}⊙(1+G_{mask})+B_{corr}$$

This element-wise gating functions as a localized amplifier or attenuator, where $G_{mask}$ determines the sensitivity of a sensor node’s reaction to the control signal based on its current state, and $B_{corr}$ provides a direct magnitude shift. By coupling future setpoints with historical momentum through spatial propagation and gated modulation, this mechanism translates discrete operational commands into continuous system-level thermodynamic responses, bridging predictive modeling and proactive process optimization.

### 4.5. Loss Function

To ensure high numerical precision while effectively tracking unsteady dynamic trends, we design a composite objective function. This approach aims to simultaneously ensure robustness against industrial noise and mitigate the phase lag and over-smoothing issues inherent in forecasting non-stationary processes. Specifically, Mean Absolute Error (MAE) is selected as the primary loss. Complementing this, we introduce a temporal gradient regularization term that explicitly penalizes the error of first-order temporal differences, effectively mitigating phase lag and enhancing the model’s response to rapid regime switches. The global optimization objective is defined as:

(23) $$L=\frac{1}{N\times Q}\sum_{b,i,t}\left(\underset{\mathrm{Value}\;\mathrm{Fidelity}}{\underset{︸}{\left|{\hat{y}}_{b,i,t}-y_{b,i,t}\right|}}+\lambda \underset{\mathrm{Trend}\;\mathrm{Consistency}}{\underset{︸}{\left|\nabla_{t}{\hat{y}}_{b,i,t}-\nabla_{t}y_{b,i,t}\right|}}\right)$$

Here, i indexes the sensor nodes, ensuring the model optimizes the global state of the entire sintering system. $\nabla_{t}$ denotes the temporal difference operator, and λ is a hyperparameter used to trade-off between value fidelity and trend consistency.

## 5. Experiment

### 5.1. Datasets and Preprocessing

#### 5.1.1. Dataset Description

The real-world datasets were acquired from the DCS of two distinct sintering production lines, designated as Machine #1 and Machine #2, at a large-scale steel enterprise. These datasets, denoted as Sinter-A and Sinter-B, cover the full process workflow spanning from raw material batching, mixing, sintering, and flue gas circulation. They encompass a comprehensive set of critical process variables, including material feed rates, water addition flow, ignition information, wind box temperatures, and exhaust gas composition, along with relevant control parameters. Table 1 summarizes the basic statistics.

To connect the mathematical framework in Section 3 with the industrial environment, graph nodes are partitioned according to the physical workflow of the DCS. Table 2 summarizes the variable categories across five primary subsystems: material proportioning, mixing and moisture control, ignition and gas supply, the strand and bellows, and the exhaust and circulation system. Although the two plants differ in scale and instrumentation, variables in both datasets are organized within a uniform functional framework. The set of exogenous control setpoints, $V_{ctrl}\subset V$, comprises input variables adjusted directly by operators, such as proportioning ratios, valve commands, and fan speeds. Since these setpoints are pre-scheduled, their future values are deterministic and known beforehand. Target state variables, $V_{target}\subset V$, consist of endogenous outputs that require continuous monitoring but cannot be directly manipulated. These indicators span a wide range of process parameters, including moisture content, ignition temperatures, wind box thermal and pressure states, and flue gas compositions, as well as various flow and temperature measurements across the circulation branches. Accurately forecasting these variables is fundamental to process control, as they serve as proxies for sinter quality and fuel consumption. Other auxiliary measurements, primarily mechanical feedback signals designated as Process Value (PV) in the DCS, serve as contextual state nodes to provide spatial context during graph message passing, though they are masked during loss calculation.

While both production lines generally operate under stable conditions, they exhibit significant differences in scale, sensor layout, and data distribution, providing two distinct testing environments. Sinter-A (Standard Scenario) was collected from Machine #1 with 183 nodes. The sensor layout is basic, primarily monitoring temperature and pressure at key locations with limited flow monitoring. Due to the concentrated numerical distribution and relatively stable operating conditions, this dataset serves as a benchmark for evaluating prediction accuracy in standard industrial scenarios; Sinter-B (High-Variance Scenario) was collected from the larger-scale Machine #2 with 221 nodes. Compared to Sinter-A, this dataset includes dense flow monitoring in the return air branch and multiple CO concentration detection nodes. Due to the physical characteristics of the return air system, these sensors exhibit high flow rates and high fluctuations. This introduces significant variance and local high-amplitude noise into the dataset, imposing higher requirements on the model’s robustness and noise tolerance.

#### 5.1.2. Data Preprocessing

To address multivariable coupling and inherent transport delays in the sintering process, we constructed the train samples using the following strategies:

Sliding Window Generation. We set both the historical observation horizon *P* and the future prediction horizon Q to 12 time steps. With a five-minute sampling interval, this corresponds to one hour, which covers a complete cycle of the primary gas–solid coupling thermal reactions in the sintering strand. To separate past states from future control intentions, we represent a data sample as two matrices.

The historical raw input $X_{t-P+1:t}\in {\mathbb{R}}^{P\times N\times D_{in}}$ captures sensor states over the past P steps. The feature dimension $D_{in}=K+2$ is formed by concatenating the one-dimensional local node state, K globally broadcasted driving factors, and a one-dimensional temporal encoding. The future control matrix $C_{t+1:t+Q}\in {\mathbb{R}}^{Q\times N\times D_{fut}}$. provides known exogenous inputs for the next Q steps. To keep a consistent N node graph structure matching historical observations, we zero-pad the unobserved features of non-control nodes. Here, $D_{fut}=K+2$ consists of known local exogenous control setpoints ($v_{i}\in V_{ctrl}$) or masked values, the K global factors, and future temporal encodings.

Global Feature Broadcasting: Since key global variables govern the macroscopic dynamics of the sintering strand, we replicate them across the spatial dimension to match the N nodes and concatenate them with local features. Based on the specific physical layouts and control systems, the global variable sets (K) are defined as follows:

- For Sinter-A (K=7): Strand speed command, circulation fan speed command, ignition gas valve command, combustion air valve command, primary and secondary mixing water valve command, and dynamically calculated total feed rate.
- For Sinter-B (K=5): Strand speed command, circulation fan frequency command, ignition gas valve command, combustion air valve command, and the dynamically calculated total feed rate.

To prevent data leakage, we compute the total feed rate dynamically by aggregating deterministic bin scale commands instead of using physical weighbridge measurements. As all K global variables are scheduled exogenous commands $V_{ctrl}$ within the DCS, their future trajectories over horizon Q are fully predetermined before inference.

Temporal Encodings: To capture periodic operational patterns, we apply a normalized diurnal time encoding ($D_{time}=1$) to both historical and future matrices. Each timestamp is mapped linearly into the interval [0,1) based on its fractional elapsed time within a 24 h cycle. This continuous scalar representation enables the network to recognize daily production cycles and shift-change patterns.

The prediction objective ${\hat{Y}}_{t+1:t+Q}\in {\mathbb{R}}^{Q\times N_{target}\times 1}$ is defined as the actual process values of selectively target nodes $V_{target}$ over the future Q steps. This ensures the model focuses entirely on the critical endogenous indicators required for process stability analysis.

#### 5.1.3. Process-Based Physical Prior Graph

To explicitly represent material flow and transmission delays, we pre-calculate the physical prior adjacency matrix $A_{prior}$ for model initialization using a Gaussian kernel. This kernel relies on the equivalent process lag $d_{i,j}$ between nodes i and j, empirically derived from stage-wise transit times and domain knowledge:

(24) $$A_{i,j}=\left\{\begin{array}{ll} \exp\left(-\frac{d_{i,j}^{2}}{\sigma^{2}}\right), & \mathrm{if}\;\exp\left(-\frac{d_{i,j}^{2}}{\sigma^{2}}\right)\geq \epsilon \\ 0, & \mathrm{otherwise} \end{array}\right.$$

The attenuation coefficient σ acts as a macroscopic thermodynamic time constant. Given the plant’s nominal strand speed of 1.5 to 2.5 m per minute, we set σ=250. Physically, this ensures the correlation weight between variables separated by a primary heat transfer delay of 250 s (approximately 4.1 min) decays to $e^{-1}\approx 0.36$. To prevent spurious noise propagation from weakly related remote sensors, we apply a sparsity threshold of ϵ=0.001. This truncates edges with a process lag exceeding roughly 11 min ($d_{i,j}>656\;s$), effectively decoupling them. We also enforce self-loops to preserve the historical feature evolution of each node.

### 5.2. Experimental Setup

Normalization Strategy: To address the dimensional heterogeneity and non-stationary characteristics of sintering data, a two-stage normalization approach was implemented. Node-wise Z-Score normalization was applied to the input data. To ensure a fair comparison and mitigate distribution drifts under unsteady conditions, the Reversible Instance Normalization (RevIN) [36] module was uniformly integrated into all models. This module dynamically removes non-stationarity during forward propagation and restores the original physical distribution via learnable affine transformations at the output.

Data Splitting and Statistics: To prevent temporal data leakage, the datasets were partitioned chronologically into training, validation, and testing sets with a ratio of 7:1:2. Table 3 summarizes the detailed statistical properties across these splits for both production lines. The statistical metrics, such as the mean and standard deviation of temperatures and gas concentrations, exhibit noticeable variations across the splits. These variations highlight the inherent distribution shifts and non-stationary characteristics across different operational periods of the sintering process, validating the necessity of our integrated normalization strategies.

Training Strategy: The model is optimized using AdamW with a weight decay of $1\times 10^{-4}$ to enhance regularization in the noisy sintering environment. Following the established strategy of spatiotemporal benchmarks ([10,32]), we set the batch size to 32 and the initial learning rate to $1\times 10^{-3}$, which optimally balances gradient stability and hardware efficiency. To prevent overfitting, a 15-epoch early stopping mechanism is employed to tolerate transient validation loss fluctuations. All reported results represent the average of five independent runs with random initializations to ensure statistical reliability.

Parameters Setup: In the experiments conducted on all datasets, the parameters listed in Table 4 are used.

Evaluation Metrics: To comprehensively quantify model performance, RMSE and MAE were used to measure the absolute deviation of predictions. Additionally, Mean Absolute Percentage Error (MAPE) and Weighted Mean Absolute Percentage Error (WAPE) were employed to evaluate relative errors. In addition, WAPE was adopted to address the significant magnitude differences inherent in industrial data. Unlike MAPE, which computes the simple average of percentage errors, WAPE weights the error by the actual value summation, preventing the overall evaluation from being skewed or diluted by sensors with vastly different scales.

(25) $$\mathrm{RMSE}=\sqrt{\frac{1}{N\cdot Q}\sum_{i,t}{\left({\hat{y}}_{i,t}-y_{i,t}\right)}^{2}}$$

(26) $$\mathrm{MAE}=\frac{1}{N\cdot Q}\sum_{i,t}|{\hat{y}}_{i,t}-y_{i,t}|$$

(27) $$\mathrm{MAPE}=\frac{1}{N\cdot Q}\sum_{i,t}\left|\frac{{\hat{y}}_{i,t}-y_{i,t}}{y_{i,t}}\right|\times 100\%$$

(28) $$\mathrm{WAPE}=\frac{\sum_{i=1}^{N}|y_{i}-{\hat{y}}_{i}|}{\sum_{i=1}^{N}|y_{i}|}\times 100\%$$

### 5.3. Performance Evaluation

To rigorously evaluate the performance of ProMix-DGNet, we compared it against three categories of baseline methods, ranging from traditional statistical approaches to state-of-the-art spatiotemporal neural networks:

- Statistical Baselines: Historical Average (HA) and Historical Inertia (HI).
- Basic Deep Learning Baselines: LSTM, Transformer [37], DLinear [33], iTransformer [34], and MLP.
- STGNNs: AGCRN [32], DGCRN [38], Graph WaveNet [10], DST-SGNN [39] and TGGC [40].

To ensure a fair comparison regarding the utilization of future control information, all baseline models were equipped with a linear projection layer to process the future control matrix $C_{t+1:t+Q}$, forming a standardized benchmark against the Future Control-Informed Mechanism explicitly designed in our model. Table 5 summarizes the performance metrics, and Table 6 presents the statistical significance tests across five independent runs.

Overall, ProMix-DGNet achieves the lowest prediction errors on most absolute evaluation metrics. When evaluated against recent spatiotemporal baselines, the model outperforms DST-SGNN across all metrics on both datasets. The comparison with TGGC presents a more nuanced picture. On the relatively stable Sinter-A dataset, TGGC holds a marginal edge in RMSE and MAPE, while ProMix-DGNet leads in MAE and WAPE. On the highly volatile Sinter-B dataset, however, our model outperforms TGGC by over 2.3% in both MAE and RMSE, and leads in WAPE. This indicates that while TGGC is optimized for capturing stable periodic trends, our architecture is more resilient to severe operational fluctuations and large-scale outliers. To verify that these performance divergences are not artifacts of random initialization, paired *t*-tests confirm the statistical distinctiveness between the models. The improvements over strong baselines on Sinter-B yield p<0.001, indicating that the resilience to extreme industrial noise is driven by the architectural design rather than stochastic variance.

Although deep learning models consistently outperform statistical methods, recent attention-based models such as iTransformer show suboptimal performance. This discrepancy stems from two factors. Modern transformers are predominantly optimized for long-term forecasting by extracting macroscopic periodicities, whereas this short-horizon task (P=12,Q=12) requires immediate responsiveness to localized thermodynamic perturbations and operational commands. Furthermore, without spatial prior constraints, unconstrained global attention mechanisms struggle to preserve the strict sequentiality of the sintering strand. They are susceptible to capturing spurious correlations from high-frequency sensor noise, which supports the necessity of a structured process-view global mixer and physical graph anchoring.

The Sinter-B dataset reveals an evaluation discrepancy in industrial forecasting where the HI method, defined as ${\hat{Y}}_{t+1}=Y_{t}$, yields a low MAPE of 9.31% alongside a high RMSE of 1093.17. This difference arises from a scale bias, where the large numerical magnitudes of industrial variables dilute continuous one-step lag errors into small percentage deviations. The outlier-sensitive RMSE, however, penalizes these substantial absolute deviations, indicating the inability of heuristic methods to anticipate system dynamics. Unlike naive heuristics, deep learning models learn non-linear transitions to predict future states. ProMix-DGNet achieves the lowest overall RMSE of 952.83. By filtering noise through the decoupled two-stream topology, the model proactively anticipates dynamic evolution and mitigates the lagging effects inherent in simpler methods.

### 5.4. Ablation Study

#### 5.4.1. Analysis of Core Modules

We constructed three variants by removing key components: w/o Mixer (removing Process-View Global Mixer), w/o BiLSTM (removing future control guidance), and w/o Dynamic (removing dynamic graph constructor). The results are summarized in Table 7.

Process-View Global Mixer is the primary driver for performance under high-volatility conditions. On the Sinter-B dataset, removing this module leads to a sharp increase in RMSE, from 952.83 to 985.78 (+32.95). This confirms that for large-scale, fluctuating industrial data, local graph convolutions alone are insufficient to capture the macroscopic thermodynamic field. Conversely, on the stable Sinter-A dataset, the impact is negligible, with an RMSE of 59.59 compared to 59.47 for the variant. This suggests that local dependencies suffice for steady-state prediction, whereas the mixer’s expansive receptive field becomes critical only when the system deviates from equilibrium.

Dynamic Graph Constructor enhances precision in stationary scenarios. On Sinter-A, its removal causes the RMSE to increase from 59.59 to 61.47. In stable regimes, the model relies on precise topological drifts to capture subtle operational variations. On the noisy Sinter-B dataset, the gain is less pronounced (952.83 vs. 954.72), as environmental noise can partially mask the benefits of dynamic edge evolution.

Future control guidance provides consistent, albeit auxiliary, performance gains across both datasets. In Sinter-B, the inclusion of this module reduces RMSE by approximately 2.82. Integrating the future control matrix $C_{t:t+Q}$ allows the model to leverage known setpoints as constraints to account for physical control lags and suppress extreme prediction outliers.

#### 5.4.2. Analysis of Component Variants

As shown in Table 8, we evaluate different implementations for the three core functional layers, where the Decoding Encoder column refers to the BiLSTM-based future control guidance component described in Section 4.4.

To address architectural choices compared to models like TSMixer, the spatial-dimension MLP (Process-View Global Mixer) was evaluated against a channel-mixing MLP and a spatial self-attention variant. The channel-mixing variant, which processes the internal features of each sensor independently, exhibits significant degradation on the Sinter-B dataset, with the RMSE increasing to 985.67. Standard channel mixers are spatially blind, failing to model cross-sensor thermodynamic propagation across the sintering strand. The temporal mixer commonly used in TSMixer is not adopted here because temporal dependencies are already modeled by the Gated TCN.

A spatial MLP is more process-aware than standard attention mechanisms in industrial contexts. As evidenced by the spatial attention variant, which yields an RMSE of 981.43 on Sinter-B, attention-based models are sensitive to high-frequency industrial noise. Attention mechanisms rely on data-driven, dynamic weight allocation that transient sensor anomalies can easily bias, leading to unstable representations. In contrast, the spatial MLP learns a structured, global transformation matrix that functions as a stable parametric prior of the entire sintering process. By enabling deterministic all-to-all interactions through a shared parameter matrix, the spatial MLP anchors the global thermodynamic field, making it more robust against localized sensor noise than the dynamic weight reassignment of self-attention.

Comparing the RBF kernel with dot-product attention highlights the robustness of the topological modeling. On Sinter-A, the RBF-based graph and dot-product attention yield nearly identical results, with RMSE values of 59.59 and 59.37. On the high-variance Sinter-B dataset, the RBF kernel achieves a lower RMSE of 952.83 compared to 957.77 for dot-product attention. Dot-product attention computes unbounded similarity weights sensitive to magnitude outliers, whereas the RBF kernel evaluates absolute Euclidean distances. This introduces an exponential decay effect that provides a robust representation by truncating the influence of abnormal spatial deviations under extreme conditions.

To evaluate the decoding mechanism, the BiLSTM-based module for future control guidance was replaced with a standard Transformer encoder. As shown in Table 8, the Transformer yields a slight RMSE increase on the Sinter-B dataset (953.76 vs. 952.83) and offers no informational advantage for this auxiliary task. Within our architecture, this decoding step serves as a targeted feature-processing link rather than the primary backbone. Embedding a Transformer, which possesses a quadratic computational complexity of $O(L^{2})$, introduces structural redundancy without performance gains. Thus, the BiLSTM-based design provides an efficient trade-off for real-time industrial deployment.

### 5.5. Parameter Sensitivity Analysis

To assess the robustness of ProMix-DGNet, we conducted a sensitivity analysis on three hyperparameters: the sparsity threshold of the dynamic graph (*k*), the node embedding dimension of the adaptive static graph ($D_{d}$), and the expansion ratio of the process-view global mixer (*r*).

#### 5.5.1. Impact of Dynamic Sparsity Threshold

**Figure 2:**
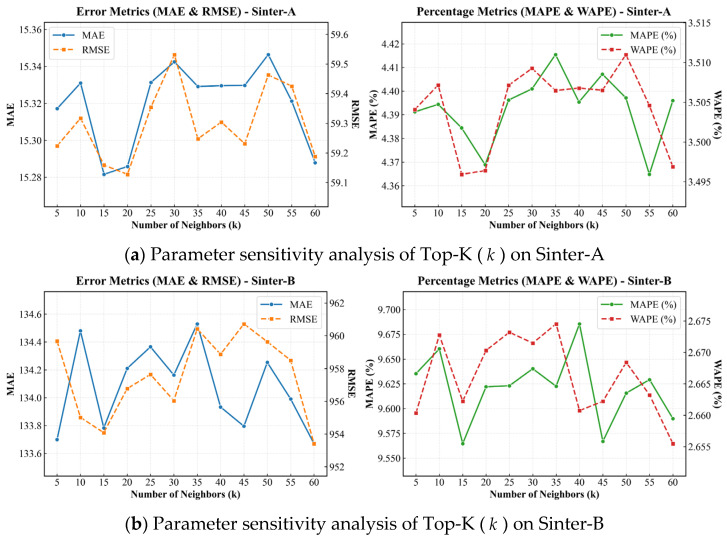
Parameter sensitivity analysis of Top-K (k).

The sparsity threshold *k* determines the receptive field size during the dynamic graph learning process. As illustrated in Figure 2, evaluating the top-*k* neighbors within a continuous range of k∈[5,60] reveals distinct behaviors across datasets. On the stable Sinter-A dataset (Figure 2a), the model benefits from a broader neighborhood, with the error curves reaching a minimum RMSE of 59.13 and an MAE of 15.29 at k=20. When the threshold is restricted to k=10, the model lacks sufficient topological information, causing the RMSE to rise to 59.32. In environments with lower noise interference, aggregating broader contextual information enables the detection of subtle, global flow field dynamics.

For the high-variance Sinter-B dataset (Figure 2b), the model achieves optimal performance with a sparser topology, reaching an RMSE of 954.08 at k=15. Expanding the neighborhood size to k=45 deteriorates the RMSE to 960.73. A large dynamic neighborhood introduces spurious connections, propagating high-amplitude, localized noise across the network. A smaller threshold, such as k=15, truncates these noisy connections to balance structural information aggregation and noise suppression.

#### 5.5.2. Impact of Static Graph Embedding Dimension

**Figure 3:**
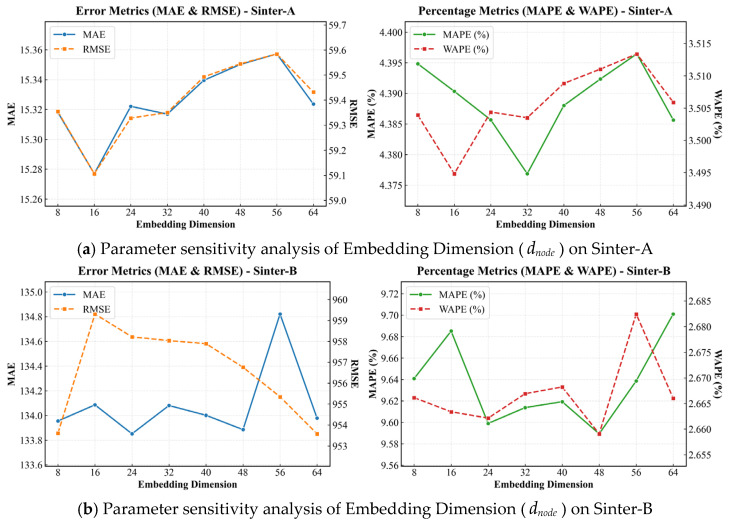
Parameter sensitivity analysis of Embedding Dimension ($d_{node}$).

The node embedding dimension $D_{d}$ determines the rank and feature capacity of the learnable static adjacency matrix, affecting the encoding of time-invariant structural priors. Figure 3 illustrates performance variations across different embedding dimensions. On the stable Sinter-A dataset (Figure 3a), the model favors compact representations, with the error curves reaching a minimum at $D_{d}=16$, achieving an RMSE of 59.11 and an MAE of 15.28. Restricting the dimension to $D_{d}=8$ provides insufficient capacity to reconstruct the physical topology, increasing the RMSE to 59.36. Increasing the embedding dimension beyond 16 on Sinter-A degrades performance, with the RMSE rising to 59.58 at $D_{d}=56$. For processes with stable thermodynamic conditions, high-dimensional embeddings introduce parameter redundancy, causing the adaptive graph to memorize transient noise rather than structural dependencies.

For the Sinter-B dataset (Figure 3b), an evaluation trade-off emerges. The RMSE is lowest at $D_{d}=64$, while the MAE reaches its optimum at $D_{d}=24$. Because $D_{d}=24$ reduces the parameter count by approximately 62.5% compared to $D_{d}=64$ while maintaining competitive accuracy, a moderately compact embedding is sufficient to capture stable physical connections in high-variance scenarios without introducing computational redundancy.

#### 5.5.3. Impact of Expansion Ratio in Process-View Global Mixer

**Figure 4:**
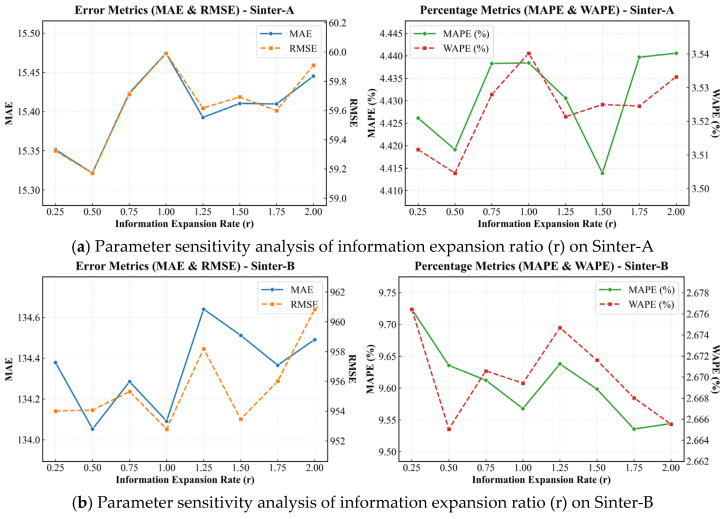
Parameter sensitivity analysis of information expansion ratio (r).

The expansion ratio *r* controls the hidden layer dimensionality within the spatial MLP of Process-View Global Mixer, dictating the trade-off between non-linear representation capacity and the risk of overfitting. Figure 4 illustrates performance variations across a range of r∈[0.25,2.0]. On the stable Sinter-A dataset (Figure 4a), the error curves reach a minimum at a bottleneck configuration of r=0.5, achieving an RMSE of 59.17 and an MAE of 15.32. Increasing the expansion ratio beyond this point degrades performance, with the RMSE rising to 59.99 at r=1.0. Under stable thermodynamic conditions, compressing the spatial dimension allows the global mixer to function as a spatial filter, forcing the network to discard redundant localized noise and retain macroscopic physical constraints.

Conversely, the Sinter-B dataset requires a higher representation capacity. As shown in Figure 4b, the optimal RMSE shifts to r=1.0 (952.79), with an MAE of 134.09. Given the denser sensor layout and localized fluctuations in Sinter-B, a dimension-preserving latent space maps long-range variable couplings without information loss. Expanding the network capacity diminishes performance across both datasets; for example, the RMSE on Sinter-B peaks at 960.85 when r=2.0. High model complexity provides degrees of freedom that cause the spatial MLP to overfit high-frequency industrial noise rather than learning the heat and mass transfer laws.

### 5.6. Practicality and Interpretability Analysis

#### 5.6.1. Robustness Under Distinct Noise Regimes

**Figure 5:**
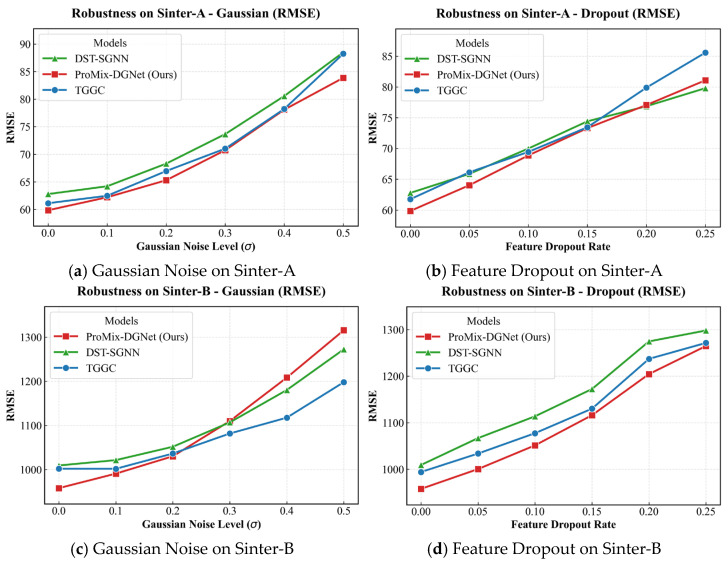
Robustness evaluation measured by RMSE across Sinter-A and Sinter-B datasets under Gaussian noise and feature dropout.

To validate the resilience of ProMix-DGNet against real-world industrial anomalies, we conducted stress tests on the Sinter-A and Sinter-B datasets. We compared the pre-trained model with TGGC and DST-SGNN by applying perturbations strictly to the test set during inference. As illustrated in Figure 5, we simulated two industrial scenarios: random feature dropout, masking a fraction p∈[0.05,0.25] of historical sensor inputs to zero to simulate sensor malfunctions and data transmission loss; and Gaussian noise injection, adding a noise distribution $N(0,\sigma^{2})$ where σ∈[0.1,0.5] into normalized inputs to simulate high-frequency measurement fluctuations in dusty or high-temperature environments.

As shown in Figure 5b,d, ProMix-DGNet demonstrates resilience to sensor dropout. On the high-variance Sinter-B dataset, even at an extreme 25% dropout rate, the model maintains a lower RMSE of 1259.02 compared to 1271.49 for TGGC and 1298.17 for DST-SGNN. This performance stems from structural redundancy and decoding anchors. The learnable static physical graph and the process-view global mixer compensate for degraded dynamic topologies by incorporating macroscopic process context. When individual sensor data is lost, the global mixer leverages correlations from the entire sintering strand. Furthermore, the scheduled future control variables $C_{t:t+Q}$ act as noise-free deterministic anchors during the decoding stage. These variables prevent forecasting divergence when historical observations are corrupted, ensuring the model remains aligned with the intended operational trajectory.

Under typical industrial Gaussian noise ranges (σ≤0.3), the model maintains a lower error rate. As shown in Figure 5a,c, under extreme theoretical noise (σ≥0.4), the degradation slope steepens, and the model is eventually surpassed by cosine-similarity-based baselines such as TGGC. This phenomenon highlights a mathematical boundary of the dynamic graph driven by RBF. Extreme high-amplitude white noise distorts multi-dimensional Euclidean distances, which form the basis of the RBF-driven topological construction, causing the learned topology to collapse under extreme variance. Models based on cosine similarity are less sensitive to pure magnitude distortions because they focus on the orientation of feature vectors. Despite this theoretical boundary, extreme independent noise where σ≥0.4 rarely occurs in practical DCS without triggering safety alarms or signal isolation. The performance of ProMix-DGNet within realistic anomaly boundaries (σ≤0.3) confirms its practical robustness for industrial deployment.

#### 5.6.2. Case Study and Error Diagnosis

To complement aggregate metrics with interpretable evidence, two representative regimes commonly observed in industrial sintering systems are analyzed: dynamic thermal transitions and impulse-like measurement corruption.

**Figure 6:**
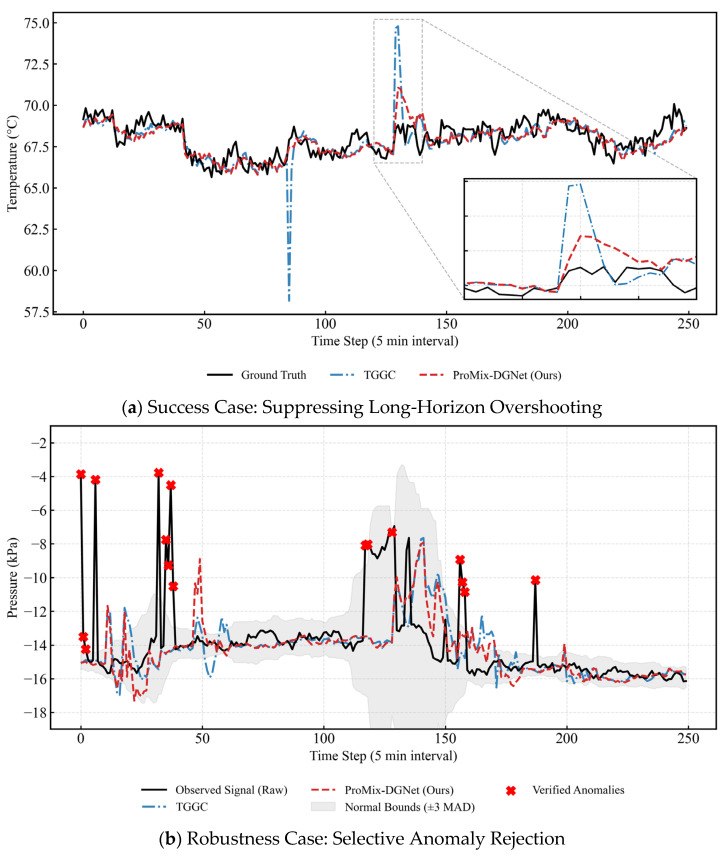
Systematic qualitative diagnosis of ProMix-DGNet across two critical scenarios.

Figure 6a illustrates a temperature node undergoing a rapid thermal transition. In this scenario, TGGC generates a sharp transient spike in the highlighted region, peaking at 74.66 °C, which exceeds the local variation envelope and deviates from the underlying trend. This phenomenon represents a typical long-horizon extrapolation artifact when handling high-gradient shifts. In contrast, ProMix-DGNet remains closely aligned with the true physical trajectory, anchoring at 69.35 °C, and attenuates the overshoot magnitude. This stability under dynamic transitions is consistent with the future control-informed decoding design, indicating a reduced tendency to produce non-physical extremes under multi-step error propagation.

Figure 6b presents a pressure signal characterized by severe impulse-like spikes in the raw observed readings. These anomalies are independently verified using a Hampel/Median Absolute Deviation (MAD) rule with ±3 MAD bounds. Around and following these anomaly bursts, TGGC exhibits stronger oscillatory responses and delayed echo behaviors, where historical noise propagates into future horizons. Conversely, ProMix-DGNet avoids tracking isolated impulses and maintains a trajectory closer to the nominal regime, achieving an 18.7% MAE reduction in the post-anomaly window. This mitigation of delayed artifacts benefits from the spatial-view MLP, providing improved robustness to localized measurement corruption without propagating historical noise into future predictions.

#### 5.6.3. Computational Complexity and Real-Time Inference Feasibility

To evaluate the integration of ProMix-DGNet into existing DCS, its computational complexity and execution latency were profiled. Profiled using the thop library, ProMix-DGNet requires 0.41 million parameters and 8.12 GFLOPs for the Sinter-A configuration with 183 nodes. For the larger Sinter-B configuration with 221 nodes, it scales to 0.69 million parameters and 12.51 GFLOPs. This demonstrates the O(N) linear scalability of the process-view global mixer, avoiding the $O(N^{2})$ parameter explosion typical of standard spatial self-attention networks.

In real-world sintering plants, macro-control loops typically operate with an update frequency of 1 to 5 min. The end-to-end execution latency was evaluated under a standard rolling-horizon operation with a batch size of 32. The GPU inference latency is 264.28 ms for Sinter-A and 337.87 ms for Sinter-B. Even when accounting for data I/O overheads, such as fetching approximately 2200 float values via Open Platform Communications Unified Architecture (OPC UA) protocols and performing local normalization (typically taking < 200 ms), the entire execution completes in under one second.

This sub-second latency enables decentralized edge deployment, demonstrating a cloud-training and edge-inference asymmetric hardware requirement. Training the spatiotemporal network from scratch requires high-performance GPUs due to backpropagation across dynamic graphs, but the inference phase is efficient. The architecture can be compiled via Open Neural Network Exchange (ONNX) and deployed onto industrial ARM-based edge SoCs equipped with Neural Processing Units (NPU), such as the Rockchip RK3588S providing 6 TOPS. In a DCS topology, low-power edge gateways can be installed alongside Programmable Logic Controllers (PLCs). By retrieving high-frequency data via the OPC-UA protocol and executing millisecond-level inference locally, this decentralized edge-deployment paradigm eliminates the communication latency and privacy risks associated with cloud-based inference, satisfying the real-time computing requirements of industrial closed-loop control.

## 6. Conclusions

This study proposes a Process-aware Mixed Dynamic Graph Network (ProMix-DGNet) for sintering system prediction. By leveraging global feature broadcasting and input projection, the model explicitly encodes the multivariable coupling between process-level boundary conditions and local sensor data. We effectively capture the spatiotemporal correlations through a Decoupled Two-Stream Topology Learning mechanism and a Process-View Global Mixer, which are designed to model both adaptive dynamic dependencies and long-range process coupling. Furthermore, we introduce a Future Control-Informed Module to dynamically integrate known future control setpoints, enhancing the model’s ability to adapt to non-stationary condition drifts. Extensive experiments on two real-world industrial datasets (Sinter-A and Sinter-B) demonstrate the competitive performance of ProMix-DGNet. Moving forward, we plan to explore the use of generative models to infer potential control intentions when signals are missing. we aim to embed this model into reinforcement learning frameworks to achieve closed-loop intelligent regulation of the sintering process.

## Figures and Tables

**Table 1:** Basic information of the dataset.

Dataset	Time Range	Nodes	Edges	Time Step	Sampling Period
Sinter-A	27 November 2024~16 January 2025	183	1182	15,582	5 min
Sinter-B	20 July 2025~10 September 2025	221	1047	14,529	5 min

**Table 2:** Categorization and Mapping of Physical Variables in Datasets.

Physical Subsystem	Node Category	Variable Identifiers $(\mathrm{Sinter}\text{-}A,\;N_{A}=183$)	Variable Identifiers $(\mathrm{Sinter}\text{-}B,\;N_{B}=221$)
Material Proportioning	$\mathrm{Exogenous}\;\mathrm{Control}\;V_{ctrl}$	Proportion_Ratio_1 to 16 Bin_Scale_CMD_1 to 16	Proportion_Ratio_1 to 19 Bin_Scale_CMD_1 to 19
	Contextual State	Bin_Scale_PV_1 to 16	Bin_Scale_PV_1 to 19
Mixing & Moisture	$\mathrm{Exogenous}\;\mathrm{Control}\;V_{ctrl}$	Pri/Sec_Mix_Water_Valve_CMD	None
	$\mathrm{Target}\;\mathrm{State}\;V_{target}$	$\mathrm{Pri}/\mathrm{Sec}\_\mathrm{Mix}\_\mathrm{Water}\_V_{ctrl}$ Flow Pri/Sec_Mix_Water_Press	Pri/Sec_Mix_Water_Flow Pri/Sec_Mix_Moisture Pri_Mix_Water_Press/Temp
	Contextual Stat	Mix_Water_Valve_PV_1/2 Mix_Water_Valve_PV Mixed_Bin_Steam_Flow Mixed/Hearth_Bin_Weight	Mixed_Bin_Steam_State Mixed/Hearth_Bin_Weight
Ignition & Gas	$\mathrm{Exogenous}\;\mathrm{Control}\;V_{ctrl}$	Gas_Valve_CMD Comb_Air_Valve_CMD Total_Air_Valve_CMD	Gas_Valve_CMD Comb_Air_Valve_CMD Comb_Oxygen_Valve_CMD_1/2
	$\mathrm{Target}\;\mathrm{State}\;V_{target}$	Ignition_Furnace_Temp_B/N Ignition_Gas_Flow/Press Comb_Air_Flow Comb_Fan_Press	Ignition_Furnace_Temp_B/N Ignition_Gas_Flow/Press/Temp Comb_Air/Oxygen_State Enriched_Oxygen_Flow/Rate
	Contextual Stat	Gas/Air_Valve_PV	Ignition_Gas/Oxygen_Valve_PV
Strand & Bellows	$\mathrm{Exogenous}\;\mathrm{Control}\;V_{ctrl}$	Strand_Speed_CMD Branch_Valve_State_4...19	Strand_Speed_CMD Roller_Feeder_Speed_CMD Branch_Valve_State_4...18
	$\mathrm{Target}\;\mathrm{State}\;V_{target}$	Windbox_Temp_1 to 24 Windbox_Press_1 to 24 Windbox_Branch_Flow_4...19	Windbox_Temp_1...23 Windbox_Press_1...23 Windbox_Branch_Flow
	Contextual Stat	Strand_Speed_PV/State Material_Thickness_N/S	Strand_Speed/Feeder_PV Surface_Steam_State
Exhaust & Circulation	$\mathrm{Exogenous}\;\mathrm{Control}\;V_{ctrl}$	Circulation_Fan_Damper_CMD Circulation_Fan_Speed_CMD Hood_Damper_CMD_1...18	Circulation_Fan_Freq_CMD Circulation_Damper_CMD Hood_Return_Damper_CMD_7...12
	$\mathrm{Target}\;\mathrm{State}\;V_{target}$	Main_Exhaust_CO Main_Exhaust_O2 Circulation_Flue_Press/Flow/Temp	Windbox_CO_4/7 Return_Main_CO Main_Exhaust_CO/O2 Circulation_Fan_Press/Temp Main_Flue_Temp/Press Hood_Return_Flow_1 to 12
	Contextual Stat	Circulation_Fan_Damper/Speed_PV Hood_Damper_PV_1...18	Circulation_Fan_Freq/Damper_PV Hood_Return_Damper_PV_7 to 12

**Table 3:** Statistical properties across data splits for Sinter-A and Sinter-B.

Physical Property	Sinter-A (Standard)						Sinter-B (High-Variance)					
	Train Set		Val Set		Test Set		Train Set		Val Set		Test Set	
	Mean	STD	Mean	STD	Mean	STD	Mean	STD	Mean	STD	Mean	STD
Temperature (°C)	212.66	289.58	206.89	288.7	215.25	296.52	212.31	242.37	201.95	238.13	220.30	246.72
Pressure/Draft (KPa)	15.13	108.16	21.6	160.47	15.1	103.39	−12.76	45.25	−11.32	41.70	−13.12	42.39
Flow/Volume (m^3^/h)	1537	2560	1522	2585	1563	2570	18,419	15,262	16,758	14,400	16,980	13,758
Weight/Ratio	43.15	167.26	42.83	162.66	43.23	167.03	22.14	31.19	20.74	26.10	21.74	26.67
Gas Conc. (ppm/%)	1885.22	1901.69	1908.74	1913.79	1930.5	1940.94	3960.96	2972.58	4078.26	3112.07	5151.84	3387.17
Others (Valve/Speed)	44	36.93	43.55	36.35	44.18	36.94	30.44	38.70	28.69	36.61	27.60	36.18

**Table 4:** Architectural Hyperparameters of ProMix-DGNet.

Hyperparameter	Sinter-A	Sinter-B
Input Feature Dimension ($D_{in}$)	9	8
Hidden Channel Dimension ($D_{h}$)	32	32
Spatiotemporal Encoder Layers (L)	4	4
Prediction Horizon (Q)	12	12
Dilation Rates ($d_{l}$)	1, 2, 4, 8	1, 2, 4, 8
Sparsity Threshold (Top-k)	20	25
Initial Node Embedding Dimension ($D_{d}$)	40	45
Encoder Expansion Ratio (r)	1.0	1.0
BiLSTM Hidden Size	32	32

**Table 5:** Performance Comparison of Different Models on the Sinter-A and Sinter-B Datasets.

Dataset	Sinter-A				Sinter-B			
Model	MAE	RMSE	MAPE (%)	WAPE (%)	MAE	RMSE	MAPE (%)	WAPE (%)
HA	48.018	154.996	9.981	9.544	741.190	2356.967	22.900	13.436
HI	17.161	66.149	4.862	3.934	142.218	1093.168	**9.3113**	2.8819
LSTM	15.994	61.581	4.750	3.658	138.293	1023.685	9.814	**2.622**
Transformer	16.115	62.323	4.752	3.687	143.184	1065.538	10.183	2.656
iTransformer	16.125	62.336	4.762	3.690	143.725	1077.140	10.207	2.640
DLinear	16.226	62.723	4.776	3.715	142.844	1007.854	9.864	2.787
MLP	16.169	62.403	4.817	3.700	159.303	1067.816	11.242	3.057
AGCRN	15.755	60.547	4.381	3.605	144.254	1019.581	9.980	2.869
DGCRN	16.745	65.019	4.889	3.828	141.603	1021.329	10.060	2.808
GWNet	15.737	60.714	4.663	3.601	135.505	995.881	9.792	2.696
DST-SGNN	16.233	62.755	4.776	3.716	142.726	1008.164	9.756	2.785
TGGC	15.379	**59.195**	**4.313**	3.518	137.090	976.166	9.360	2.735
**Ours**	**15.354**	59.589	4.397	**3.512**	**133.856**	**952.833**	9.622	2.656

Bold: best result in each column. Underline: second-best result. Results averaged over 5 runs.

**Table 6:** Statistical Significance Test between ProMix-DGNet and Key Baselines.

Dataset	Metric	GWNet	DST-SGNN	TGGC (SOTA)	Ours	*p*-Value	Imp. (%)
Sinter-A	MAE	15.737 ± 0.069	15.354 ± 0.048	16.233 ± 0.051	15.379 ± 0.117	0.546	0.16%
	RMSE	60.714 ± 0.412	59.589 ± 0.421	62.755 ± 0.148	59.195 ± 0.575	0.142	−0.67%
	MAPE (%)	4.663 ± 0.044	4.397 ± 0.018	4.776 ± 0.011	4.313 ± 0.022	<0.001	−1.95%
	WAPE (%)	3.601 ± 0.016	3.512 ± 0.011	3.716 ± 0.012	3.518 ± 0.029	0.5898	0.15%
Sinter-B	MAE	135.505 ± 1.149	133.856 ± 0.556	142.726 ± 0.650	137.090 ± 0.433	<0.001	2.36%
	RMSE	995.881 ± 13.870	952.832 ± 1.525	1008.164 ± 0.869	976.166 ± 6.549	<0.001	2.39%
	MAPE (%)	9.792 ± 0.094	9.623 ± 0.084	9.756 ± 0.056	9.360 ± 0.089	0.0014	−2.81%
	WAPE (%)	2.695 ± 0.023	2.656 ± 0.016	2.785 ± 0.005	2.735 ± 0.007	<0.001	2.86%

**Table 7:** Ablation study results of ProMix-DGNet on different datasets.

Dataset	Model	MAE	RMSE	MAPE (%)	WAPE (%)
Sinter-A	w/o Mixer	15.375	59.473	4.418	3.517
	w/o BiLSTM	15.369	59.509	4.398	3.516
	w/o Dynamic	15.880	61.474	4.678	3.634
	Ours	15.354	59.589	4.397	3.512
Sinter-B	w/o Mixer	138.886	985.776	9.835	2.727
	w/o BiLSTM	134.549	955.650	9.563	2.678
	w/o Dynamic	133.909	954.719	9.604	2.663
	Ours	133.856	952.833	9.622	2.656

**Table 8:** Ablation study of component variants on Sinter-A and Sinter-B datasets.

Dataset	Variant	Global Mixer	Dynamic Graph	Decoding Encoder	MAE	RMSE	MAPE (%)	WAPE (%)
Sinter-A	w/Channel-only	Channel-only	RBF	BiLSTM	15.849	61.567	4.590	3.628
	w/SpatialAttn	Attn.	RBF	BiLSTM	15.913	61.785	4.663	3.641
	w/Dot-Product	Spatial MLP	Dot-Product	BiLSTM	15.319	59.366	4.384	3.504
	w/Transformer	Spatial MLP	RBF	Transformer	15.332	59.544	4.388	3.507
	Ours	Spatial MLP	RBF	BiLSTM	15.354	59.589	4.397	3.512
Sinter-B	w/Channel-only	Channel-only	RBF	BiLSTM	137.874	985.669	9.774	2.71
	w/SpatialAttn	Attn.	RBF	BiLSTM	136.707	981.425	9.720	2.694
	w/Dot-Product	Spatial MLP	Dot-Product	BiLSTM	134.040	957.770	9.620	2.666
	w/Transformer	Spatial MLP	RBF	Transformer	133.831	953.755	9.598	2.660
	Ours	Spatial MLP	RBF	BiLSTM	133.856	952.833	9.622	2.656

## Data Availability

Data are available from the corresponding author upon reasonable request. Public access is restricted due to commercial confidentiality and privacy concerns related to the enterprise’s industrial Distributed Control System.
